# Isolation and Structural Characterization of Bioactive Molecules on Prostate Cancer from Mayan Traditional Medicinal Plants

**DOI:** 10.3390/ph11030078

**Published:** 2018-08-14

**Authors:** Rafael Sebastián Fort, Juan M. Trinidad Barnech, Juliette Dourron, Marcos Colazzo, Francisco J. Aguirre-Crespo, María Ana Duhagon, Guzmán Álvarez

**Affiliations:** 1 Laboratorio de Interacciones Moleculares, Facultad de Ciencias, Universidad de la República, Montevideo, C.P. 11400, Uruguay; rfort@fcien.edu.uy (R.S.F.); juan.manuel.trinidad13@gmail.com (J.M.T.B.); 2 Laboratorio de Moléculas Bioactivas, CENUR Litoral Norte, Universidad de la República, Ruta 3 (km 363), Paysandú, C.P. 60000, Uruguay; juli.dourron@gmail.com; 3 Departamento de Química del Litoral, CENUR Litoral Norte, Universidad de la República, Paysandú, C.P. 60000, Uruguay; mcolazzo@gmail.com; 4 Facultad de Ciencias Químico Biológicas, Universidad Autónoma de Campeche, Campeche, C.P. 24039, Mexico; fjaguirr@uacam.mx; 5 Departamento de Genética, Facultad de Medicina, Universidad de la República, Montevideo, C.P. 11800, Uruguay

**Keywords:** prostate cancer, in vitro, LNCaP, natural product, plants, Mayan medicine

## Abstract

Prostate cancer is the most common cancer in men around the world. It is a complex and heterogeneous disease in which androgens and their receptors play a crucial role in the progression and development. The current treatment for prostate cancer is a combination of surgery, hormone therapy, radiation and chemotherapy. Therapeutic agents commonly used in the clinic include steroidal and non-steroidal anti-androgens, such as cyproterone acetate, bicalutamide and enzalutamide. These few agents have multiple adverse effects and are not 100% effective. Several plant compounds and mixtures, including grape seed polyphenol extracts, lycopene and tomato preparations, soy isoflavones, and green tea extracts, have been shown to be effective against prostate cancer cell growth. In vivo activity of some isolated compounds like capsaicin and curcumin was reported in prostate cancer murine models. We prepared a library of plant extracts from traditional Mayan medicine. These plants were selected for their use in the contemporaneous Mayan communities for the treatment of different diseases. The extracts were assessed in a phenotypic screening using LNCaP prostate cancer androgen sensitive cell line, with a fixed dose of 25 μg/mL. MTT assay identified seven out of ten plants with interesting anti-neoplastic activity. Extracts from these plants were subjected to a bioguided fractionation to study their major components. We identified three compounds with anti-neoplastic effects against LNCaP cells, one of which shows selectivity for neoplastic compared to benign cells.

## 1. Introduction

Currently, there are many millenary cultures such as the Mayans who use plants to treat diverse human diseases. In some areas of Mexico, large communities of people are only treated by their traditional physicians (called “Chamanes”) who reach some success using traditional herbal medicine [1]. Although many plants are used for different purposes, scientific support for their application is still required. Due to the frequent consumption of these plants, there are many reports on their good toxicology that warrant their human use [2,3]. The search for natural products for cancer therapy represents an area of great interest in which plants have been the most important source. As an example, different plant extracts and herbal compounds studied in traditional Chinese medicine showed promising anti-prostate cancer activity [4]. Indeed, many clinically successful anti-cancer drugs are either natural products themselves or have been developed from natural occurring lead compounds [5,6]. One example used in the therapy of prostate cancer (PCa) is docetaxel [7]. Despite their different mechanisms of action, most of these compounds exhibit cytotoxic activity which is useful in cancer therapy where the goal is to kill cancerous cells. 

Cancer remains one of the major causes of mortality worldwide and PCa is the most common cancer among males in Western countries [8]. PCa is a complex heterogeneous disease which shows a heterogeneous prognosis [9]. The current treatment for PCa is a combination of surgery, radiation, and chemotherapy. The therapeutic agents commonly used in the first line of PCa treatment include steroidal (cyproterone acetate) and nonsteroidal anti-androgens (like bicalutamide and enzalutamide) [10,11,12]. Steroids have partial agonistic activity and effects that extend to other hormonal systems, leading to many complications, including serious cardiovascular problems, gynecomastia, loss of libido and erectile dysfunction [10,13]. Non-steroidal anti-androgens also show several side effects, but have an improved oral bioavailability which favors their use over steroidal anti-androgens [12,14].

In the present context, there is a need for new more efficient and specific PCa drugs causing less severe side effects. Successful strategies for drug discovery employ the phenotypic screening of neoplastic cells [15]. Seeking for the discovery of anti-neoplastic compounds, the present investigation evaluate the anti-neoplastic activity of extracts from *Cnidoscolus chayamansa* McVaugh (Euphorbiaceae), *Byrsonima crassifolia* L. Kunth (Malpighiaceae)*, Leucaena leucocephala* Lam. de Wit (Fabaceae), *Malmea depressa* Baillon R.E. Fries (Annonaceae), *Ipomoea pes-caprae* L. R. Br. (Convolvulaceae)*, Capsicum chinense* Jacq. (Solanaceae)*, Terminalia catappa* L. (Combretaceae)*, Helicteres baruensis* Jacq. (Sterculiaceae), *Cecropia obstusifolia* Bertol. (Moraceae) and *Coccoloba uvifera* L. (Polygonaceae) and identifies active compounds using the PCa cell line LNCaP. The isolated compounds were structurally characterized by GC/MS and NMR analysis. 

**Figure 1:**
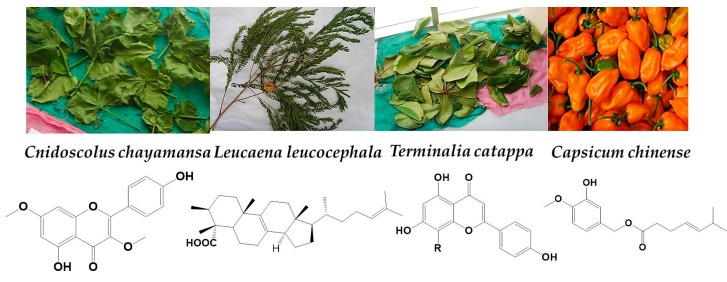
Pictures of plants assayed in this study and derived compounds previously reported in the literature [18,20,21,22,23].

*C. chayamansa*, commonly known as “chaya” (Figure 1) is extensively used in Mexican daily diet due to its high nutritional value, but its anti-tumor activity has not been studied so far. However, it is known to display anti-mutagenic activity in the bacterial cell lines TA100 and TA98 [16]. In addition, ischemia-reperfusion tests performed in mice showed its anti-inflammatory and anti-oxidant activity in the ethanolic extract [16,17,18]. These data suggests its potential prophylactic and therapeutic use in cancer treatment [19].

*B. crassifolia* is a native American orchid species whose leaves produce a drug named birsonimadiol, an anti-inflammatory agent that suppresses the production of nitric oxide (NO) and prostaglandin E_2_ (PGE_2_), decreasing the gene expression of cyclooxygenase-2 (COX-2), tumor necrosis factor alpha (TNF-alpha) and interleukin 6 (IL-6) in lipopolysaccharide-stimulated macrophages [24]. Since IL-6 is an important determinant of PCa progression [25], the organic extract of *B. crassifolia* constitutes an interesting candidate for PCa treatment. 

*L. leucocephala,* known as “huaje” by the Mayan culture, displays anti-proliferative effects in the cell lines HT-29 (human colon carcinoma), HeLa (human cervical carcinoma), HepG3 (human liver carcinoma) and MCF-7 (human breast adenocarcinoma), being the last one the most affected by the ethanolic extract. These effects are attributed to its promising condensed tannin compounds, which potentially hold health promoting qualities [26]. In addition, *L. leucocephala* extracts showed cytotoxic activity in SCC9 and SAS cells [20], while some of its chemical components have significant chemo-preventive and anti-proliferative properties [27].

*M. depressa*, also known as “elemuy”, was reported to inhibit the growth of HT-29 (human colon carcinoma), MCF-7 (human breast carcinoma) and A-549 (human lung carcinoma) cell lines [28]. Mice trials performed with *I. pes-caprae* in melanoma (B16F10) resulted in a decrease in tumor growth that position it as a complementary treatment to radiotherapy [26].

*T. catappa*, commonly referred as “almendro”, is a tropical and subtropical plant widely used in folk medicine. Since the ethanolic extract obtained from its leaves decreases the invasion of metastatic cells in oral and lung cancer cell lines [22,29], it is an interesting plant to further study. In addition, ethanol extracts of *Terminalia catappa* leaves were shown to exhibit an anti-tumor effect against Ehrlich ascites carcinoma by modulating lipid peroxidation [30]. 

*C. uvifera,* known as “niiche” or “Uva de mar”, displays anti-oxidant and anti-tyrosinase activities and also inhibites the production of IL-1alpha, TNF-alpha and alpha-MSH in melanocytes subjected to UV radiation [31]. 

*C. chinense*, called “chile habanero”, is one of the spiciest chilies of the genus, being widely used as a spicy sauce in Mexican food. Some of its compounds have been tested in clinical trials [32,33,34]. Capsaicin is the major components of *C. chinense* responsible for the burning sensation in the spices of the genus *Capsicum* spp. and is a well-known anti-neoplastic molecule in PCa and others tumors [34,35,36]; indeed, it has a proven anti-proliferative effect against the LNCaP cell line in a time and concentration dependent manner [37]. Thus, we decided to use *C. chinense* as a positive control for the bioguided fractionation. According to the NIH, there are four molecules derived from this kind of plants in clinical trials linked to cancer (two completed and two in progress) [38,39]. 

The only plant present in the study that does not present literature associated with cancer is *H. baruensis,* commonly referred as “tsutsup”. These families of plants have some toxicological reports and anti-parasitic activity reported, but nothing specific to *H. baruensis* [40,41].

## 2. Results and Discussion

### 2.1. Plant Collection and Extract Preparation

Based on prescription frequency, we selected 10 plants from southeastern Mexico (Caribbean region) of more than 300 species used in traditional Mayan medical practice (Table 1). These plants were chosen according to indications of the “chamanes” (the traditional Mayan doctors) and were then classified taxonomically by botanists. Although Mayan doctors recommend the use of these plants to treat human affections, the precision of the diagnosis is hampered by the absence of conventional medical consultation and treatment decisions that largely rely on religious ideas. In this context, to avoid bias in the process, the selection of the plants was independent from the disease in which they are used.

A robust system was used to maximize the extraction of stable molecules. Methanol was used to extract the most hydrosoluble compounds and dichloromethane was used to extract the most lipophilic components. High temperature was used in some steps to enhance solubilization. As shown in Table 1, extraction yield was appropriate to be applied in drug development process considering the good accessibility to the plant material and the simplicity of the instrument and methods used. 

### 2.2. Bioguided Fractionation

**Figure 2:**
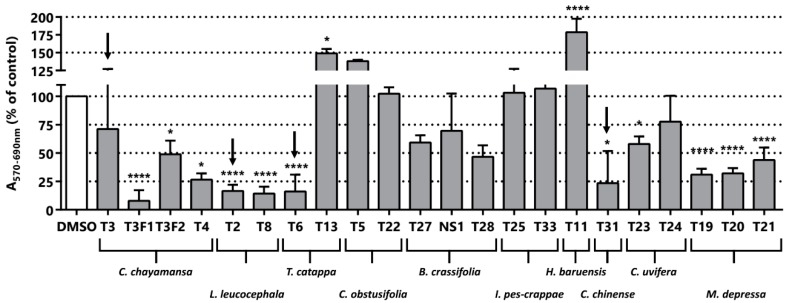
Phenotypic screening. Cytotoxic/anti-proliferative activity of the extracts (25 µg/mL) in LNCaP cells. Black arrows indicate samples selected for the next step of the bioguided fractionation procedure. The nomenclature of the fractions corresponds to a code of the preparation procedure. T3F1 and T3F2 fractions of *C. chayamansa* are derived from the sugar extraction of the methanolic extract with ethyl acetate/water. NS1 it is a solid derived from the T27 fraction isolated by filtration during the evaporation process. Statistical significance is * *p* < 0.05; **** *p* < 0.0001.

The cytotoxic/anti-proliferative activity of the 22 fractions derived from the 10 plants was evaluated in the LNCaP cell line by MTT assays. We obtained the methanolic extract, the dichloromethane extract and some solid material from the filtration of the precipitated material mentioned. Dried plant extracts were dissolved in dimethylsulfoxide (DMSO) and used at a fixed dose of 25 µg/mL in the MTT assay. Putative MTT false positives due to direct reduction of MTT by remnant extract compounds were excluded studying the effect of the extracts on MTT absorbance in the absence of cells [42]. Although, some extracts caused a reduction of MTT, its magnitude is negligible in comparison to the reduction of MTT caused by the cells treated with them (Figure S1). In addition, we carried out a microscopic inspection of the cultures after the treatment to record alterations in cell number and morphology indicative of changes in cell proliferation and cell viability/death respectively. We found that reduction in MTT was always accompanied by changes in either cell number or morphology, which indicates that the reduction of MTT was not caused by a sole change in mitochondrial activity. As shown in Figure 2, we found seven plants whose extracts display in vitro anti-neoplastic activity in LNCaP. *C. obstusifolia*, *I. pes-caprae* and *H. baruensis Jacg* extracts were not cytotoxic/anti-proliferative at the tested concentration. Strikingly, some extracts showed increased activity in the MTT assay compared to the control, which is indicative of a pro-proliferative activity and/or a boosting effect on mitochondrial metabolism (T13, T5 and T11). Among the seven plants with cytotoxic/anti-proliferative activity in LNCaP (Figure 2), *C. chayamansa* (T3F1, T3F2 and T4), *L. leucocephala* (T2 and T8), *T. catappa* (T6), *B. crassifolia* (T28), *C. chinense* (T31), *M. depressa* (T19, T20 and T21) altered MTT absorbance greater than a 50%. Moreover, this threshold is reached by all the fractions from *L. leucocephala* and *M. depressa* showed cytotoxic/anti-proliferative greater than 50% (Figure 2). As expected, *C. chinense* (T31) showed a cytotoxic/anti-proliferative activity that validates this step of the fractionation. 

**Figure 3:**
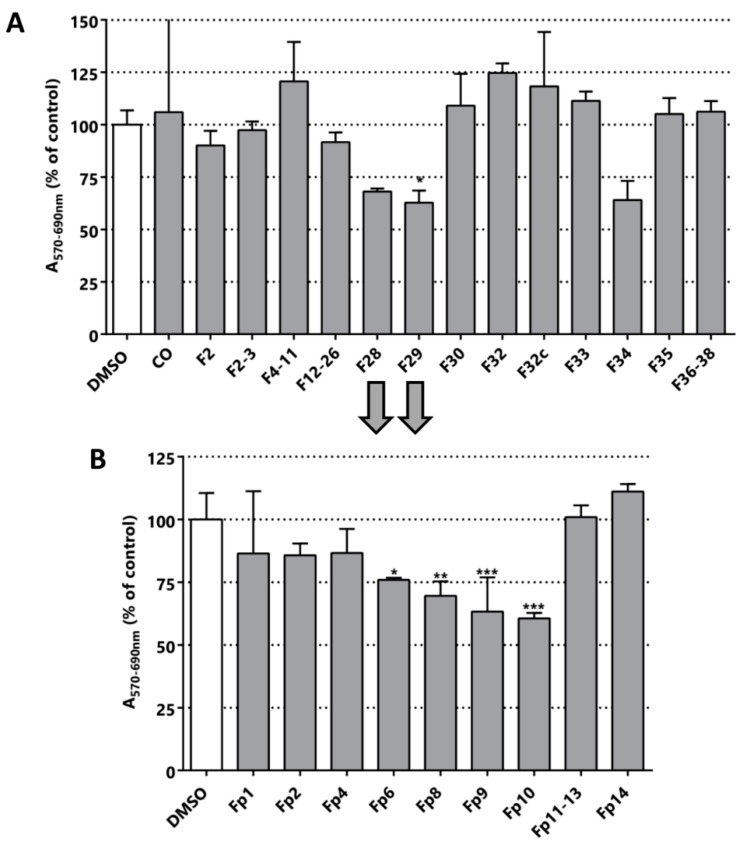
Bioguided fractionation. Cytotoxic/anti-proliferative activity of the *L. leucocephala* extracts (25 µg/mL) in LNCaP cells. (**A**) Evaluation of the fractions obtained from the silica chromatography of the methanolic extract of *L. leucocephala*. CO is the organic phase after the extraction with ethyl acetate/water. (**B**) Evaluation of the fractions obtained by preparative chromatography, particularly fractions F28 and F29. The nomenclature of the fractions corresponds to the chromatographic elution time. Statistical significance is * *p* < 0.05; ** *p* < 0.01; *** *p* < 0.001.

Four active extracts were selected to continue the bioguided fractionation (blacks arrows in Figure 2 indicate the selected samples). The selected extracts comprise the methanolic extracts from *L. leucocephala* (T2), *C. chayamansa* (T3), *T. catappa* (T6) and *C. chinense* (T31). The new fractions derived from the fractionation by silica chromatography of the methanolic extract of *L. leucocephala* were again evaluated in LNCaP cells using the MTT assay. However, the MTT activity of these sub-fractions did not improve and even worsened in this purification stage (Figure 3A). The fractions F28, F29 and F34 were the most active fractions of *L. leucocephala*. The yield obtained in the first chromatography was poor and the amount of the material remaining from the next step was even lower. Indeed, there was not enough material to pursue the fractionation of F34. Nevertheless, since the TLC profile of fractions F28 and F29 were similar, they were mixed to continue with a preparative chromatography. As shown in Figure 3B, the cytotoxic/anti-proliferative effect of fraction F10 was similar to the parental fractions (preparative chromatography image available in Figure S2). However, there was not enough material to proceed to further fractionation with this extract. Although there are no reports about the activity of methanolic extracts of *L. leucocephala* in LNCaP cell line, extracts from this plant have been tested in other PCa cells, such as DU145, and some of the compounds responsible for the cytotoxic activity were structurally elucidated [26].

**Figure 4:**
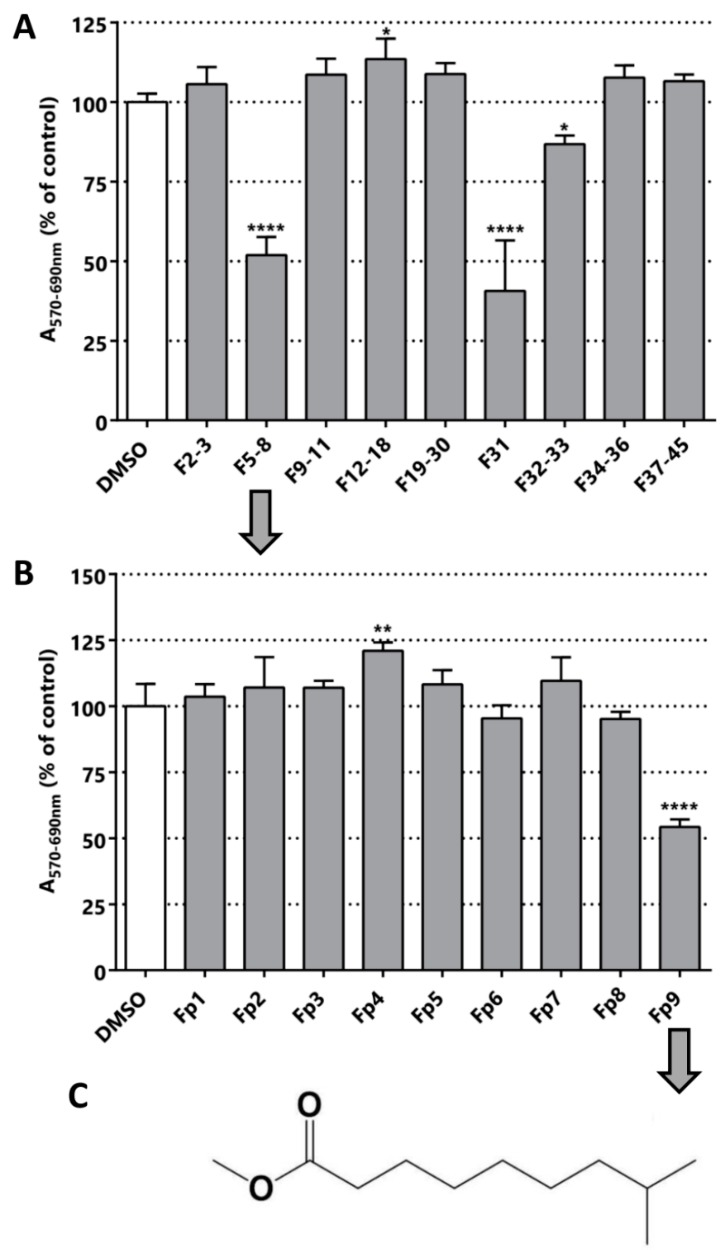
Bioguided fractionation. Cytotoxic/anti-proliferative activity of the *C. chayamansa* extracts (25 µg/mL) in LNCaP cells. (**A**) Evaluation of the fractions obtained from the silica chromatography of the methanolic extract of *C. chayamansa*. (**B**) Evaluation of the fractionations obtained by preparative chromatography of F5-8. (**C**) The structure of the compound identified in fraction Fp9: methyl ester of the fatty acid 8-methyl-6-nonanoic acid. Statistical significance is * *p* < 0.05; ** *p* < 0.01; **** *p* < 0.0001.

From the purification of T3 fraction (crude fraction from the methanolic extract of *C. chayamansa*) nine sub-fractions were obtained. Contrary to what was observed for *L. leucocephala* T2 extracts, the cytotoxic/anti-proliferative effect of fraction T3 on LNCaP was 31%, whereas the sub-fractions increased the effect to values of 48% in F5-8 and 59% in F31 (Figure 4). These results indicate that in the latter purification there was an enrichment of the active molecule(s).

The fraction F5-8 was submitted to preparative chromatography yielding nine different fractions (chromatography image available in Figure S3). Only Fp9 was active in the MTT assay. Optical microscopy images of the cultures after the treatment with this fraction and its previous fraction confirmed the cytotoxic/anti-proliferative activity determined by MTT (Figure S4). The major compound present in Fp9 fraction was then structurally characterized by NMR and MS (results available in Figures S5 and S6) and the structure is depicted in Figure 4C. The single major molecule corresponds to the methyl ester of the fatty acid 8-methyl-6-nonanoic acid (Figure 4C). Surprisingly, a relatively simple fatty acid was isolated in the form of ester, which surely derives from the successive manipulations with methanol and high temperature. The action of simple fatty acids has been reported in different types of cancer previously [43,44,45,46]. The closest related structure found in the literature was 13-methyltetradecanoic acid, which showed in vitro and in vivo anti-neoplastic activity (IC_50_ in prostate cancer cell in vitro DU145 was 62 µM) [47]. This strongly validates our bioguided fractionation procedure and opens a new class of molecules for use in a nature inspired design of new bioactive molecules for PCa. Simple fatty acids are versatile compounds that can be used in different types of reaction, as in the preparation of hybrid molecules. These molecules could be part of a new multi-target drug for PCa. 

**Figure 5:**
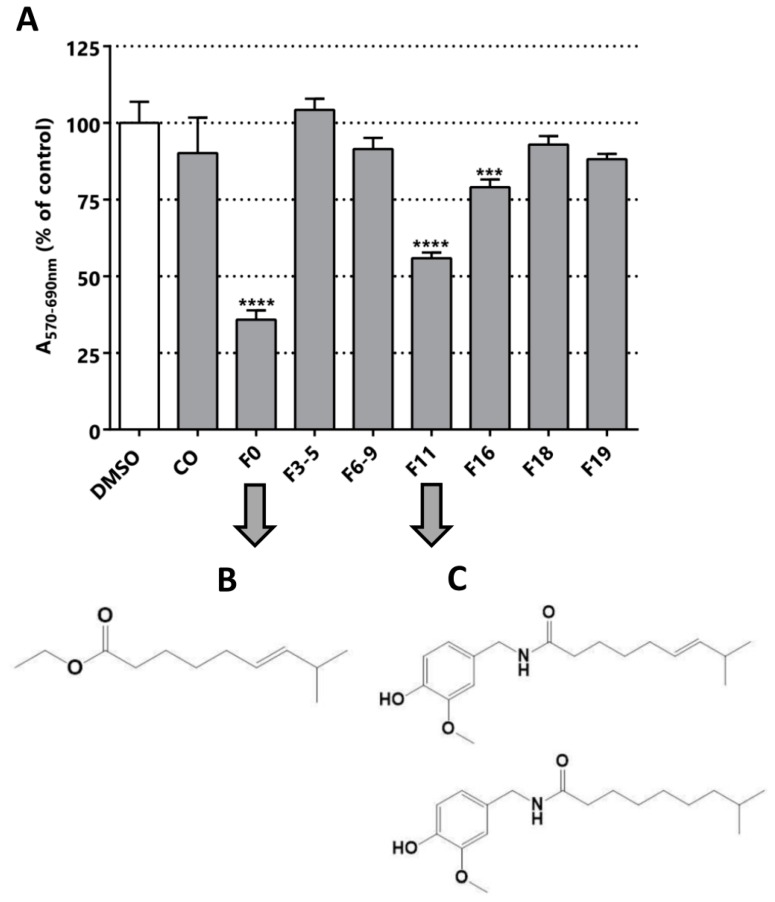
Bioguided fractionation. Cytotoxic/anti-proliferative activity of the *C. chinense* extracts (25 µg/mL) in LNCaP cells. (**A**) Evaluation of all the fractions obtained from the silica chromatography of the methanolic extract of *C. chinense*. The structure of the compounds isolated from the preparative chromatography of (**B**) F0 ((*E*)-ethyl 8-methylnon-6-enoate) and (**C**) F11 (capsaicin and dihydrocapsaicin, respectively) is shown. Statistical significance is *** *p* < 0.001; **** *p* < 0.0001.

The sub-fractionation of *C. chinense* yield seven sub-fractions, three of which were cytotoxic/anti-proliferative against the LNCaP cell line: F0, F11 and F16 (Figure 5). F11 and F16 could be similar molecules due to their elution in neighboring fractions; however, F0 is probably a different molecule. The F0 and F11 fractions were selected for further fractionation and structural elucidation, since they were the most active fractions, decreasing the MTT activity to 35% and 55% respectively (Figure 5). Optical microscopy images of the cultures after the treatment with these fractions confirmed a decrease in cell viability suggested by MTT assay (Figure S7). F0 and F11 underwent silica preparative chromatography. For F0, five different subfractions were isolated (Figure S8), among which F5p was the subfraction with the highest cytotoxic/anti-proliferative effect, reducing MTT activity to 76% (Figure S8). Despite having evidenced cytotoxic effect in LNCaP, the subsequent step of fractionation decreased the MTT effect of the subfractions. 

From the sub-fractionation of F0, we isolated the active compound (*E*)-ethyl 8-methylnon-6-enoate (Figure 5B and Figure S9). This molecule is a precursor in the biosynthesis of capsaicin, and has not been described as an inhibitor of PCa cells [32,48]. In addition, it is structurally correlated to the compound isolated from *C. chayamansa*, since it is a fatty acid with one unsaturation. Finally, we isolated a mixture of capsaicin and dihydrocapsaicin from F11 (Figure 5C and Figure S1), in a 60/40 proportion. This mixture was reported in the isolation from others types of chili [34,49]. These results confirm our bioguided procedure, because we finally purified the same product from the fruit of the *C. chinense* plant and evidenced the same range of cytotoxic/anti-proliferative effect reported previously for the LNCaP cell line [36,50]. 

### 2.3. Selectivity

**Figure 6:**
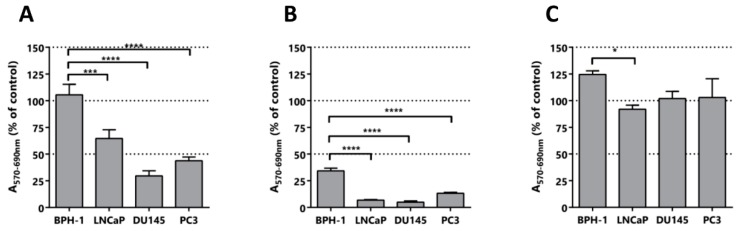
Selectivity assay. Cytotoxic/anti-proliferative activity after treatment of prostate cell lines with the indicated compounds. (**A**) (*E*)-ethyl 8-methylnon-6-enoate assayed at 100 µM, (**B**) Curcumin assayed at 50 µM and (**C**) vanillin assayed at 50 µM. The percentages of MTT activity of the compounds was calculated relative to control cultures incubated only with DMSO. Prostate cell lines: BPH-1 derived from a benign prostatic hyperplasia (control) and LNCaP, DU145 and PC3 derived from metastatic PCa. Statistical significance is * *p* < 0.05; *** *p* < 0.001; **** *p* < 0.0001.

To explore the selectivity of the isolated compounds we performed a MTT assay using different types of prostate cells lines: benign prostatic hyperplasia (BPH-1) [42], a prostate cancer cell line derived from a brain metastatic site (DU145), a prostate cancer cell line derived from bone metastasis (PC3), and a prostate cancer cell line derived from a lymph node metastasis (LNCaP). (*E*)-ethyl 8-methylnon-6-enoate was evaluated in a fixed dose of 100 μM for the 4 lines (Figure 6A). As can be seen in Figure 6, the malignant cell lines were affected in their activity, while the benign cell line was not. Remarkably, the MTT activity values for the LNCaP, DU145 and PC3 lines were 76%, 41% and 62%, respectively. This is correlated with the good toxicology profiles showed by these kind of fatty acids in other type of cells and also with their low in vivo toxicity [47]. Additionally, we tested vanillin to evaluate the possible contribution of the polyphenol motif in the anti-neoplastic activity, and curcumin, which has activity against PCa cells, as a positive control (Figure 6B). As can be observed in Figure 6C, vanillin did not affect the tested cell lines, suggesting that this pharmacophore itself is not sufficient for the cytotoxic/anti-proliferative effect observed for capsaicin. As expected, curcumin showed activity in the cell lines studied and selectivity for the neoplastic cells, but it greatly affected the benign cell line.

**Figure 7:**
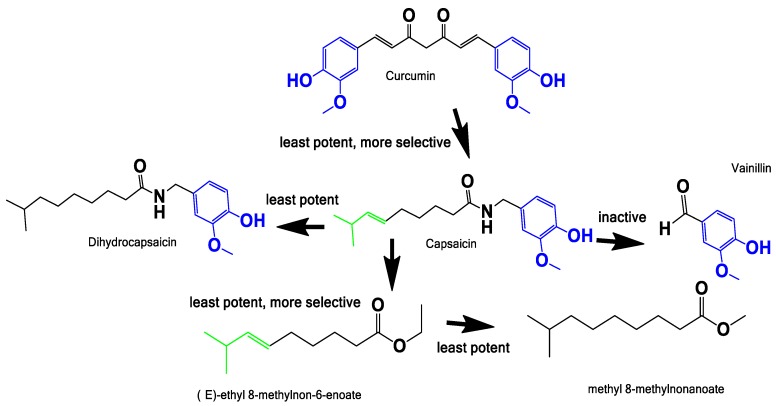
Structure activity relationship. Exploration of the possible pharmacophore responsible for the cytotoxic/anti-proliferative activities against LNCaP. Color codes indicate the vanillin motif (blue), and vinyl motif (green). The “potency” refers to the activity of the compound in LNCaP and the selectivity towards malignant vs benign cells.

Finally, the structure-activity relationship was explored to understand the relation between the vinyl and vanillin motif in the cytotoxic activity observed. We used curcumin as a known anti-neoplastic drug hit, a molecule isolated from *Curcuma longa* which is used as a food spice worldwide and has strong structural relations with the capsicum family (Figure 7). 

The curcumin was reported to be active in different cancer cell line models, infection and diseases [51,52,53,54]. Although it is labeled as a pan assay interference (PAINS) compound [55], there is a lot of toxicological information supporting its safety. Indeed, it is actually widely consumed in many diets around the world [56]. In this work, we evidenced that curcumin has similar activity in all the prostate cell models assessed (Figure 6B and Table 2). In addition, vanillin has been used as proof of the activity of the vanillin motif, and was innocuous at 100 µM (Figure 6C). Our observations and the review of the literature, allow us to propose that the region of the vanillin ring linked with the carbonyl amide bond could be an important motif involved in the cytotoxic/anti-proliferative activity. This region could be the area of the molecule that potentially interacts with the vanillin transitory receptor type 1 (TRPV1), a non-selective calcium channel involved in the burning sensation that has been proposed as one of the anti-neoplastic mechanisms of capsaicin [35,57].

The conjecture mentioned above is based on the fact that the molecules known to interact with TRPV1 are the capsaicinoids called “capsiate”, i.e., capsaicin and resiniferatoxin (RTX) [58]. They have only one common motif: the vanillin ring with the amide bond on carbon 1, where the bond can be amide (capsaicin and RTX) or ester (capsiate). TRPV1 mRNA and protein levels show a positive correlation with the level of malignancy of the tumors in PCa samples from patients, placing TRPV1 as a candidate biomarker and also a target for drugs [59]. In the LNCaP and PC-3 cell lines, this receptor is present and active, as in benign prostatic hyperplastic tissue samples, so the cell lines can be used to assays of TRPV1 activity [38]. The fatty acid motif in capsaicin seems to influence the selectivity between benign and neoplastic cells; it also has an intrinsic anti-neoplastic activity independent of the vanillin motif. Curcumin has higher activity than capsaicin, but there is not selectivity in the assayed cells. Curcumin has two vanillin motif without the fatty acid motifs, and also a Michael acceptor region, which together could explain its reduced selectivity (Figure 6). Therefore, the fatty acid is a promising motif to use in a drug design program inspired by nature. Finally, it could be useful in the design of new hybrid molecules with recognized anti-neoplastic compounds. 

## 3. Experimental Section

### 3.1. Plant Material Collection

The most popular “chamanes” in the area of Quintana Roo, where the mayor community of Mayan descendants lives, where identified. Then, these traditional doctors were interviewed to find ethnobotanic information about the plant used for disease treatment. Ten plants were selected based on the prescription frequency. The “chamanes” shared their collection spaces and helped to identify the plants. The different species of plants were classified after a botanical verification using voucher samples. The information related to the time and location of the plants collected is indicated in Table 1. An identified voucher sample has been deposited in the herbarium of the Autonomous University of Campeche.

### 3.2. Extract Preparation

The collected samples were first separated according to the part of the plant, for example, leaves, flowers fruits, etc. With this first separation we had more than one sample for each species of plant. The extracts were prepared in a classical format. After the collection, the plant material was dried in an oven at 50–60 °C for approximately twelve hours. Then the dry material was ground to powder grade in a mill (MF 10.2 Impact Grinding Head), weighed and extracted. Preferably 150 and 250 g of the plant powder were dissolved with 600–800 mL of solvent. Two types of extraction were carried out, one with methanol and the other with dichloromethane. The methanolic extract was obtained from the macerate of the plant material incubated with methanol for 4 h (600–800 mL), then the solvent was collected by filtration and a new amount of solvent was added for 4 more hours. This process was repeated four times. The same method was used to prepare the dichloromethane extract, using dichloromethane in the same way as methanol. Finally, the total methanolic and dichloromethane extract was evaporated to dryness in vacuum and the crude was stored at room temperature in hermetic bottles for later use. When two phases were observed during the evaporation process in the crude extract, an extraction was performed using ethyl acetate/water. 

### 3.3. Chromatographic Studies and Isolation of Active Constituents

The extract was adsorbed on silica gel and chromatographed on a silica gel column eluted with mixtures of petroleum ether-EtOAc-CH_2_Cl_2_ at increasing polarities. The eluted fractions were evaluated by TLC in the same condition. Elution with methanol at 10% yielded the polar material retained on the top of the column. The fractions were biological evaluated to identify the active fraction and a sub-sequent chromatography separation was made from them. The end point in some cases was an isolated compound. 

### 3.4. Determination of the Chemical Structures

Structures of isolated fractions and purified compounds were analyzed by extensive spectroscopic methods including GS-MS, UV, IR, ^1^H-NMR, ^13^C-NMR, COSY, HSQC, HMBC, DEPT, NOE-diff and NOESY experiments, using deuterated chloroform and the instrument default parameters. ^1^H- and ^13^C-NMR spectra, and the rest of the experiments (COSY, NOE-diff, HSQC, HMBC, DEPT, and NOESY) were obtained on an AVANCE DPX-400 spectrometer (Bruker Rheinstetten, Germany) at 22.16 °C. UV and IR spectra were recorded at room temperature using ACTGene-Nanodrop (Piscataway, NJ, USA) and Shimadzu (Kyoto, Japan) IR PRESTIGE-21 spectrophotometers, respectively. GC–MS analyses were performed on a HP 5890 chromatograph Series II Gas Chromatograph (Hewlett-Packard, Avondale, PA, USA) coupled to a VG Trio 2 mass spectrometer (VG Instruments, Danvers, MA, USA) using a DB-5 fused silica capillary column, 30 m × 0.25 mm i.d., 0.25 µm film thickness (J&W Scientific, Folsom, CA, USA). Injections were made in the splitless mode with a helium head pressure of 0.85 MPa (velocity: 0.35 ms^−1^). The injector, the transfer line and the source temperatures were set to 315, 300 and 220 °C respectively. The temperature program was: 80 °C for 1 min followed by a gradient at 4 °C min^−1^ to 300 °C, final temperature held for 30 min. Scan rate was adjusted to 1s per scan from 50 to 700 amu. The “non-aromatic hydrocarbon” fraction was dissolved in 50 µl of hexane before injection of a 1 µL aliquot. Automated Mass Spectral Deconvolution and Identification System software (AMDIS), version 2.1, provided by the National Institute of Standards and Technology (NIST, Gaithersburg, MD USA, web address: http://chemdata.nist.gov/massspc/amdis/index.html) has been used for post-processing the MS data files [60].

### 3.5. MTT Assay

BPH-1, LNCaP, PC-3 and DU145 human cell lines were obtained from the ATCC (Manassas, VA, USA). All the cell lines were maintained in RPMI 1640 (R7755) supplemented with 10% FBS (PAA™) and penicillin/streptomycin. To perform the cytotoxicity assay 1 × 10^4^ cells per well were seeded in a 96 well plate in a final volume of 200 µL of medium. The extracts or fractions were incubated for 24 h to evaluate the anti-neoplastic effect in the cell lines [61]. For selectivity assay compounds we incubated during 48 h. To proceed with the MTT assay, 20 μL of 3-(4,5-dimethylthiazol-2-yl)-2,5-diphenyl-2H-tetrazolium bromide (MTT) 5 mg/mL solution dissolved in 1× PBS was added to the wells and incubated for 4 h at 37 °C in a 5% CO_2_ controlled atmosphere. Next, the medium was aspirated,100 μL of DMSO was added to each well and the plate was incubated at room temperature for 15 min in the dark with moderate orbital shaking. Before the addition of MTT to the culture medium, all the plates were observed under the optical microscope and a semi-quantitative evaluation of changes in cell number and morphology was performed. Optical density (OD) was read on a plate spectrophotometer (Varioskan^®^ Flash Multimode, Thermo Scientific, Waltham, MA, USA) at 570 nm (for formazan absorbance measurement) and 690 nm (for background measurement) wavelengths. The methanolic and dichloromethane extracts, as well as the fractions of the chromatographies, were tested in cell lines at a fixed concentration of 25 μg/mL (on 0.3% *v*/*v* of DMSO) and, based on their activity, they were subjected to other subfractionation methods (bioguided fractionation). All the fractions were centrifuged to eliminate insoluble particles. All the analyses were done at least in triplicates. For the statistical analyses the Dunnett’s multiple comparison tests, one-way ANOVA and Student’s *t* test on GraphPad Prism 6 [62] were used. 

## 4. Conclusions

In this work, the in vitro anti-neoplastic activity of ten plants used in traditional Maya medicine were evaluated in the LNCaP cell line as a PCa biological model. *C. chayamansa*, *C. chinense*, *C. uvifera*, *L. leucocephala*, *M. depressa* and *T. catappa* showed the best cytotoxic/anti-proliferative activity. In addition, the process of bioguided fractionation of the plant extracts was standardized using the LNCaP cell line. The procedure was validated by the isolation of capsaicin from the extract of the fruit of *C. chinense*. Additionally, two novel simple fatty acids with in vitro anti-neoplastic activity were identified: 8-methyl 6-nonenoic acid ethyl ester from *C. chinense* and 8-methyl-6-nonanoic acid methyl ester from *C. chayamansa.* Finally, the selectivity for these molecules was evaluated, observing that the 8-methyl 6-nonenoic acid ethyl ester affected the malignant cells considerably more than the benign one.

## Figures and Tables

**Table 1:** Plant collection: general plant information, and extract production yields.

Common Name	Scientific Name	Location	Collection Time	Part of the Plant Used	MeOH Extract (g) *	CH_2_Cl_2_ Extract (g) *	Initial Sample (g)	Extraction Yield %
Bejuco de playa	*Ipomoea pes-caprae*	Tulum Beach (20°11′52.00′′ N; 87°26′12.38′′ O)	may-14	leaves and branches	20.2 (T25)	2.4 (T33)	132	17
Nance	*Byrsonima crassifolia*	Ecological Park Chetumal (18°30′21.25′′ N; 88°19′12.79′′ O)	sep-13	tree bark	35.5 (T27)	12.6 (T28)	200	24
Uva de mar	*Coccoloba uvifera*	Chetumal bay (18°31′1.79′′ N; 88°16′14.46′′ O)	may-14	leaves	40.0 (T24)	4 (T23)	214	21
Almendro	*Terminalia catappa*	Chetumal city (18°31′0.55′′ N; 88°18′50.27′′ O)	may-14	leaves	27.9 (T13)	9 (T6)	250	15
Elemuy	*Malmea depressa*	Santa Rosa town (19°57′51.90′′ N; 88°16′17.00′′ O)	may-14	leaves and branches	25.6 (T19)	4	164	18
Elemuy	*Malmea depressa*	Santa Rosa town (19°57′51.90′′ N; 88°16′17.00′′ O)	may-14	root	9.5 (T20)	2.6 (T21)	187	6
Huachi	*Leucaena leucocephala*	Chetumal city (18°31′17.48′′ N; 88°18′47.94′′ O)	may-14	leaves and branches	26.8 (T2)	9 (T8)	273	13
Chaya	*Cnidoscolus chayamansa*	Chetumal city (18°31′0.55′′ N; 88°18′50.27′′ O)	may-14	leaves	16.7 (T3)	2 (T4)	100	19
Guarumbo	*Cecropia obstusifolia*	Chetumal city (18°31′26.60′′ N; 88°18′49.84′′ O)	may-14	leaves	16.7 (T5)	4.5 (T22)	150	14
Tsutsup	*Helicteres baruensis Jacg*	Tulum Beach (20°11′59.42′′ N; 87°26′53.68′′ O)	may-14	leaves and branches	10.6 (T11)	6 (T14)	113	15
Chile habanero	*Capsicum chinese*	F. Carrillo Puerto town (19°34′50.49′′ N; 88° 2′39.57′′ O)	may-14	fruit	11.3 (T31)	5 (T38)	55	21

* “T#” is the Nomenclature used to name the extract prepared.

**Table 2:** IC_50_ for curcumin and (*E*)-ethyl 8-methylnon-6-enoate.

	BPH-1	DU145	PC3	LNCaP
Curcumin IC_50_ (µM)	11 ± 2	12 ± 3	8 ± 1	2 ± 1
(E)-Ethyl 8-methylnon-6-enoate at 100 µM (%) *	0	76	62	41

* % of inhibition of the cell growth at 100 µM.

## Supplementary Materials

The following are available online at http://www.mdpi.com/1424-8247/11/3/78/s1.

## References

[1] Bautista-Cruz A., Arnaud-Viñas M.R., Martínez-Gutiérrez G.A., Soledad Sánchez-Medina P., Pacheco R.P. (2011). The traditional medicinal and food uses of four plants in Oaxaca, Mexico. J. Med. Plants Res..

[2] Kumar S., Jawaid T., Dubey S. (2011). Therapeutic Plants of Ayurveda; A Review on Anticancer. Pharmacogn. J..

[3] Déciga-Campos M., Rivero-Cruz I., Arriaga-Alba M., Castañeda-Corral G., Angeles-López G.E., Navarrete A., Mata R. (2007). Acute toxicity and mutagenic activity of Mexican plants used in traditional medicine. J. Ethnopharmacol..

[4] Wang X., Fang G., Pang Y. (2018). Chinese medicines in the treatment of prostate cancer: From formulas to extracts and compounds. Nutrients.

[5] De Petrocellis L., Arroyo F.J., Orlando P., Schiano Moriello A., Vitale R.M., Amodeo P., Sánchez A., Roncero C., Bianchini G., Martín M.A. (2016). Tetrahydroisoquinoline-Derived Urea and 2,5-Diketopiperazine Derivatives as Selective Antagonists of the Transient Receptor Potential Melastatin 8 (TRPM8) Channel Receptor and Antiprostate Cancer Agents. J. Med. Chem..

[6] Hosseini A.G. (2015). Cancer therapy with phytochemicals: Evidence from clinical studies. Avicenna J. Phytomedicine.

[7] Henry J.Y., Lu L., Adams M., Meyer B., Bartlett J.B., Dalgleish A.G., Galustian C. (2012). Lenalidomide enhances the anti-prostate cancer activity of docetaxel in vitro and in vivo. Prostate.

[8] Hu Y., Fu L. (2012). Targeting cancer stem cells: A new therapy to cure cancer patients. Am. J. Cancer Res..

[9] Klarmann G.J., Hurt E.M., Mathews L.A., Zhang X., Maria A., Mistree T., Thomas S.B., Farrar W.L. (2009). Invasive Prostate Cancer Cells Are Tumor Initiating Cells That Have A Stem Cell-Like Genomic Signature. Clin Exp Metastasis.

[10] Handratta V.D., Vasaitis T.S., Njar V.C.O., Gediya L.K., Kataria R., Chopra P., Newman D., Farquhar R., Guo Z., Qiu Y. (2005). Novel C-17-heteroaryl steroidal CYP17 inhibitors/antiandrogens: Synthesis, in vitro biological activity, pharmacokinetics, and antitumor activity in the LAPC4 human prostate cancer xenograft model. J. Med. Chem..

[11] Vicentini C., Festuccia C., Angelucci A., Gravina G.L., Muzi P., Eleuterio E., Miano R., Marronaro A., Tubaro A., Bologna M. (2002). Bicalutamide dose-dependently inhibits proliferation in human prostatic carcinoma cell lines and primary cultures. Anticancer Res..

[12] Furr B.J.A., Tucker H. (1995). The preclinical development of bicalutamide: Pharmacodynamics and mechanism of action. Urology.

[13] Bobach C., Tennstedt S., Palberg K., Denkert A., Brandt W., De Meijere A., Seliger B., Wessjohann L.A. (2015). Screening of synthetic and natural product databases: Identification of novel androgens and antiandrogens. Eur. J. Med. Chem..

[14] Liedtke A.J., Adeniji A.O., Chen M., Byrns M.C., Jin Y., Christianson D.W., Marnett L.J., Penning T.M. (2013). Development of Potent and Selective Indomethacin Analogs for the Inhibition of AKR1C3 ( Type 5 17 β-Hydroxysteroid Dehydrogenase/Prostaglandin F Synthase ) in Castrate-Resistant Prostate Cancer. J. Med. Chem..

[15] Hamid R., Rotshteyn Y., Rabadi L., Parikh R., Bullock P. (2004). Comparison of alamar blue and MTT assays for high through-put screening. Toxicol. In Vitro.

[16] Loarca-Piña G., Mendoza S., Ramos-Gómez M., Reynoso R. (2010). Antioxidant, antimutagenic, and antidiabetic activities of edible leaves from *Cnidoscolus chayamansa* Mc. Vaugh. J. Food Sci..

[17] García-Rodríguez R.V., Gutiérrez-Rebolledo G.A., Méndez-Bolaina E., Sánchez-Medina A., Maldonado-Saavedra O., Domínguez-Ortiz M.Á., Vázquez-Hernández M., Muñoz-Muñiz O.D., Cruz-Sánchez J.S. (2014). *Cnidoscolus chayamansa* Mc Vaugh, an important antioxidant, anti-inflammatory and cardioprotective plant used in Mexico. J. Ethnopharmacol..

[18] Pérez-González M.Z., Gutiérrez-Rebolledo G.A., Yépez-Mulia L., Rojas-Tomé I.S., Luna-Herrera J., Jiménez-Arellanes M.A. (2017). Antiprotozoal, antimycobacterial, and anti-inflammatory evaluation of *Cnidoscolus chayamansa* (Mc Vaugh) extract and the isolated compounds. Biomed. Pharmacother..

[19] Gorrini C., Harris I.S., Mak T.W. (2013). Modulation of oxidative stress as an anticancer strategy. Nat. Rev. Drug Discov..

[20] Chung H.-H., Chen M.-K., Chang Y.-C., Yang S.-F., Lin C.-C., Lin C.-W. (2017). Inhibitory effects of *Leucaena leucocephala* on the metastasis and invasion of human oral cancer cells. Environ. Toxicol..

[21] Abu Zarin M., Wan H.Y., Isha A., Armania N. (2016). Antioxidant, antimicrobial and cytotoxic potential of condensed tannins from Leucaena leucocephala hybrid-Rendang. Food Sci. Hum. Wellness.

[22] Chu S.C., Yang S.F., Liu S.J., Kuo W.H., Chang Y.Z., Hsieh Y.S. (2007). In vitro and in vivo antimetastatic effects of Terminalia catappa L. leaves on lung cancer cells. Food Chem. Toxicol..

[23] Pino J., Sauri-Duch E., Marbot R. (2006). Changes in volatile compounds of Habanero chile pepper (*Capsicum chinense* Jack. cv. Habanero) at two ripening stages. Food Chem..

[24] Pérez Gutiérrez R.M. (2016). Anti-inflammatory effect of birsonimadiol from seeds of *Byrsonima crassifolia*. Food Sci. Biotechnol..

[25] Nguyen D.P., Li J., Tewari A.K. (2014). Inflammation and prostate cancer: The role of interleukin 6 (IL-6). BJU Int..

[26] Manigauha A., Kharya M.D., Ganesh N. (2015). In vivo antitumor potential of Ipomoea pes-caprae on melanoma cancer. Pharmacogn. Mag..

[27] She L., Liu C., Chen C., Li H., Li W., Chen C. (2017). The anti-cancer and anti-metastasis effects of phytochemical constituents from *Leucaena leucocephala*. Biomed. Res..

[28] Gutierrez-Lugo M.T., Barrientos-Benítez T., Luna B., Ramirez-Gama R.M., Bye R., Linares E., Mata R. (1996). Antimicrobial and cytotoxic activities of some crude drug extracts from Mexican medicinal plants. Phytomedicine.

[29] Yang S.F., Chen M.K., Hsieh Y.S., Yang J.S., Zavras A.I., Hsieh Y.H., Su S.C., Kao T.Y., Chen P.N., Chu S.C. (2010). Antimetastatic effects of *Terminalia catappa* L. on oral cancer via a down-regulation of metastasis-associated proteases. Food Chem. Toxicol..

[30] Tigari P., Dupadahalli K., Kamurthy H., Nadendla R., Pandya N. (2013). Antitumor and antioxidant status of *Terminalia catappa* against Ehrlich ascites carcinoma in Swiss albino mice. Indian J. Pharmacol..

[31] Silveira J.E.P.S., Pereda M.d.C.V., Eberlin S., Dieamant G.C., Di Stasi L.C. (2008). Effects of *Coccoloba uvifera* L. on UV-stimulated melanocytes. Photodermatol. Photoimmunol. Photomed..

[32] Aza-González C., Núñez-Palenius H.G., Ochoa-Alejo N. (2011). Molecular biology of capsaicinoid biosynthesis in chili pepper (*Capsicum* spp.). Plant Cell Rep..

[33] Amruthraj N.J., Raj P., Saravanan S., Lebel L.A. (2014). In vitro studies on anticancer activity of capsaicinoids from *Capsicum chinense* against human hepatocellular carcinoma cells. Int. J. Pharm. Pharm. Sci..

[34] Mori A., Lehmann S., O’Kelly J., Kumagai T., Desmond J.C., Pervan M., McBride W.H., Kizaki M., Koeffler H.P. (2006). Capsaicin, a component of red peppers, inhibits the growth of androgen-independent, p53 mutant prostate cancer cells. Cancer Res..

[35] Ziglioli F., Frattini A., Maestroni U., Dinale F., Ciuffreda M., Cortellini P. (2009). Vanilloid-mediated apoptosis in prostate cancer cells through a TRPV-1 dependent and a TRPV-1-independent mechanism. Acta Biomed. l’Ateneo Parm..

[36] Bode A.M., Dong Z. (2011). The two faces of capsaicin. Cancer Res..

[37] Ramos-Torres Á., Bort A., Morell C., Rodríguez-Henche N., Díaz-Laviada I. (2016). The pepper’s natural ingredient capsaicin induces autophagy blockage in prostate cancer cells. Oncotarget.

[38] O’Neill J., Brock C., Olesen A.E., Andresen T., Nilsson M., Dickenson A.H. (2012). Unravelling the mystery of capsaicin: A tool to understand and treat pain. Pharmacol. Rev..

[39] Rollyson W.D., Stover C.A., Brown K.C., Perry H.E., Cathryn D., Stevenson C.A.M., Ball J.G., Valentovic M.A., Dasgupta P. (2010). Bioavailability of capsaicin and its implications for drug delivery William. J. Control. Release.

[40] Balogun S.O., Da Silva I.F., Colodel E.M., De Oliveira R.G., Ascêncio S.D., De Oliveira Martins D.T. (2014). Toxicological evaluation of hydroethanolic extract of Helicteres sacarolha A. St.- Hil. et al. J. Ethnopharmacol..

[41] Mukul-Yerves J.M., Del Rosario Zapata-Escobedo M., Montes-Pérez R.C., Rodríguez-Vivas R.I., Torres-Acosta J.F. (2014). Parásitos gastrointestinales y ectoparásitos de ungulados silvestres en condiciones de vida libre y cautiverio en el trópico mexicano. Rev. Mex. Ciencias Pecu..

[42] Abel S.D.A., Baird S.K. (2018). Honey is cytotoxic towards prostate cancer cells but interacts with the MTT reagent: Considerations for the choice of cell viability assay. Food Chem..

[43] Kuhajda F.P., Jennert K., Wood F.D., Hennigart R.A., Jacobs L.B., Dick J.D., Pasternack G.R. (1994). Fatty acid synthesis: A potential selective target for antineoplastic therapy. Proc. Nati. Acad. Sci..

[44] Swinnen J.V., Roskams T., Joniau S., Van Poppel H., Oyen R., Baert L., Heyns W., Verhoeven G. (2002). Overexpression of fatty acid synthase is an early and common event in the development of prostate cancer. Int. J. Cancer.

[45] Bégin M.E., Ells G., Das U.N., Horrobin D.F. (1986). Differential killing of human carcinoma cells supplemented with n-3 and n-6 polyunsaturated fatty acids. J. Natl. Cancer Inst..

[46] De Schrijver E., Brusselmans K., Heyns W., Cells C. (2003). RNA Interference-mediated Silencing of the Fatty Acid Synthase Gene Attenuates Growth and Induces Morphological Changes and Apoptosis of LNCaP Prostate Cancer Cells. Cancer Res..

[47] Yang Z., Liu S., Chen X., Chen H., Huang M., Zheng J. (2000). Induction of Apoptotic Cell Death and in Vivo Growth Inhibition of Human Cancer Cells by a Saturated Branched-Chain Fatty Acid, 13-Methyltetradecanoic Acid. Cancer Res..

[48] Gahungu A., Ruganintwali E., Karangwa E., Zhang X., Mukunzi D. (2011). Volatile compounds and capsaicinoid content of fresh hot peppers (*Capsicum chinense*) scotch bonnet variety at red stage. Adv. J. Food Sci. Technol..

[49] Musfiroh I.D.A., Mutakin M., Angelina T., Muchtaridi M. (2013). Capsaicin level of various Capsicum fruits. Int. J. Pharm. Pharm. Sci..

[50] Laratta B., De Masi L., Sarli G., Pignone D. (2011). Hot peppers for happiness and wellness: A rich source of healthy and biologically active compounds. XV EUCARPIA Meet. Genet. Breed. Capsicum Eggplant.

[51] Anderson T.M.D. (2003). Anticancer Potential of Curcumin Preclinical and Clinical Studies. Anticancer Res..

[52] Gafner S., Lee S.K., Cuendet M., Barthélémy S., Vergnes L., Labidalle S., Mehta R.G., Boone C.W., Pezzuto J.M. (2004). Biologic evaluation of curcumin and structural derivatives in cancer chemoprevention model systems. Phytochemistry.

[53] Lin L., Shi Q., Nyarko A.K., Bastow K.F., Wu C.-C., Su C.-Y., Shih C.C.-Y., Lee K.-H. (2006). Antitumor agents. 250. Design and synthesis of new curcumin analogues as potential anti-prostate cancer agents. J. Med. Chem..

[54] Padmanaban G. (2016). Curcumin as an Adjunct Drug for Infectious Diseases. Trends Pharmacol. Sci..

[55] Wang R., Chen C., Zhang X., Zhang C., Zhong Q., Chen G., Zhang Q., Zheng S., Wang G., Chen Q.H. (2015). Structure-Activity Relationship and Pharmacokinetic Studies of 1,5-Diheteroarylpenta-1,4-dien-3-ones: A Class of Promising Curcumin-Based Anticancer Agents. J. Med. Chem..

[56] Adapala N., Chan M.M. (2008). Long-term use of an antiinflammatory, curcumin, suppressed type 1 immunity and exacerbated visceral leishmaniasis in a chronic experimental model. Lab. Invest..

[57] Kobata K., Kawaguchi M., Watanabe T. (2002). Enzymatic Synthesis of a Capsinoid by the Acylation of Vanillyl Alcohol with Fatty Acid Derivatives Catalyzed by Lipases. Biosci. Biotechnol. Biochem..

[58] Kobata K., Tate H., Iwasaki Y., Tanaka Y., Ohtsu K., Yazawa S., Watanabe T. (2008). Isolation of coniferyl esters from Capsicum baccatum L., and their enzymatic preparation and agonist activity for TRPV1. Phytochemistry.

[59] Czifra G., Varga A., Nyeste K., Marincsák R., Tóth B.I., Kovács I., Kovács L., Bíró T. (2009). Increased expressions of cannabinoid receptor-1 and transient receptor potential vanilloid-1 in human prostate carcinoma. J. Cancer Res. Clin. Oncol..

[60] Finck Y., Aydin N., Pellaton C., Gorin G., Gülaçar F. (2004). Combination of gas chromatography-mass spectrometry and mass spectral deconvolution for structural elucidation of an unusual C29-steroid detected in a complex sedimentary matrix. J. Chromatogr. A.

[61] Kawasaki B.T., Hurt E.M., Kalathur M., Duhagon M.A., John A., Kim Y.S., Farrar W.L. (2009). Effects of the sesquiterpene lactone parthenolide on prostate tumor-initiating cells: An integrated molecular profiling approach. Prostate.

[62] Fort R.S., Mathó C., Geraldo M.V., Ottati M.C., Yamashita A.S., Saito K.C., Leite K.R.M., Méndez M., Maedo N., Méndez L. (2018). Nc886 is epigenetically repressed in prostate cancer and acts as a tumor suppressor through the inhibition of cell growth. BMC Cancer.

