# Supplementary material for: Isolation and Structural Characterization of Bioactive Molecules on Prostate Cancer from Mayan Traditional Medicinal Plants
Source: Pharmaceuticals (Basel). 2018 Aug 14;11(3):78. doi: 10.3390/ph11030078 (PMC6160984; doi:10.3390/ph11030078)
Supplement: Supplementary file 1 [file pharmaceuticals-11-00078-s001.pdf]

pharmaceuticals

Supplementary Information

Figure S1.

MTT assay of the plant extracts without cells. The percentage of MTT absorbance of the extracts in the presence of LNCaP cells relative to the DMSO control (black bars) and the percentage of MTT absorbance of the cell-free extracts relative to the DMSO control (gray bars). The black arrows indicate the fractions selected for further study and the blue arrow indicates the original fraction of the compound (E)-ethyl-8-methylnon-6-enoate (tested for selectivity).

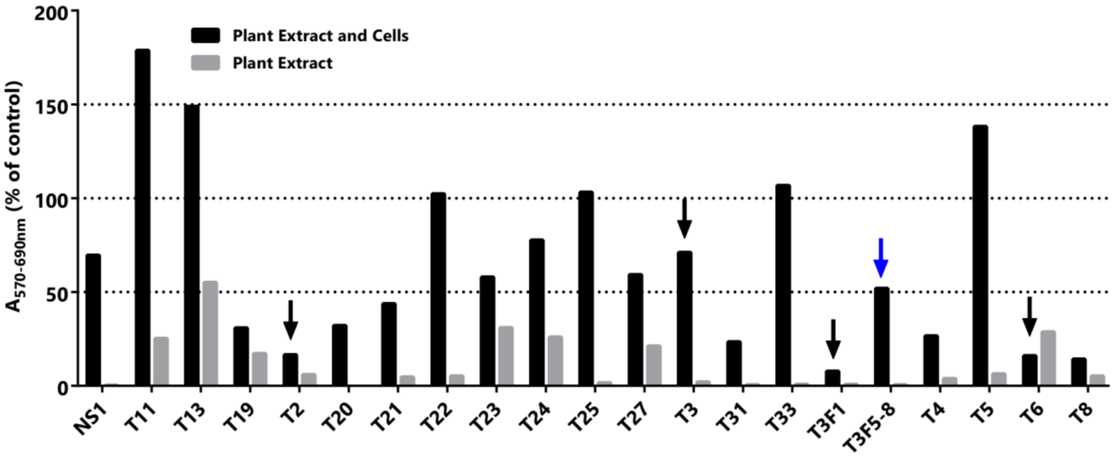

Figure S2.

Picture of the preparative chromatography of F28+F29 active fractions of *L. leucocephala* first column chromatography.

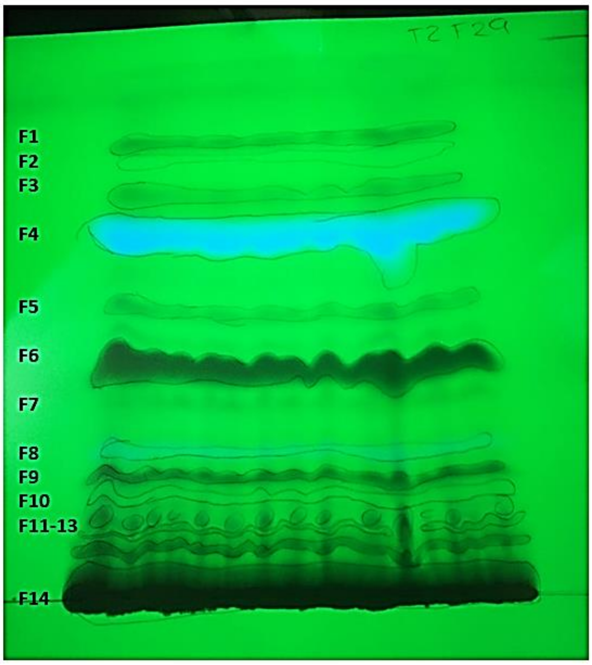

Figure S3.

Picture of the preparative chromatography of F5-8 active fractions of *C. chayamansa* first column chromatography.

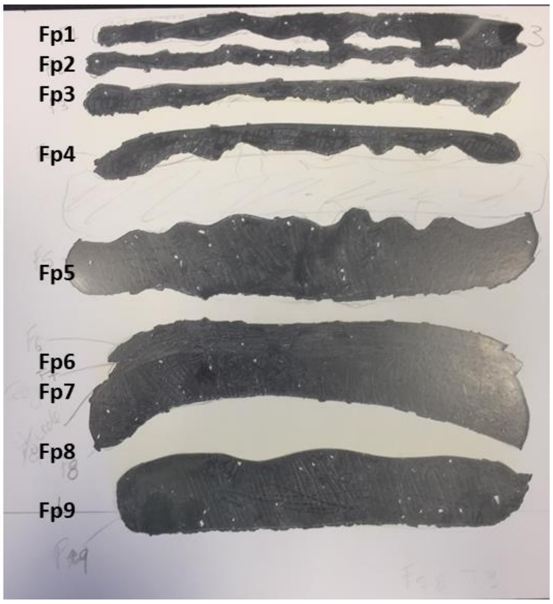

Figure S4.

Optical microscopy images of cells treated with active fractions of *C. chayamansa*: A. Representative images of optical microscopy of the LNCaP cells after treated with DMSO (20x) and F5-8 (20x and 80x) extract and previously of MTT assay. **B.** Representative images of optical microscopy of the LNCaP cells after treated with DMSO (20x) and Fp9 (20x) extract and previously of MTT assay.

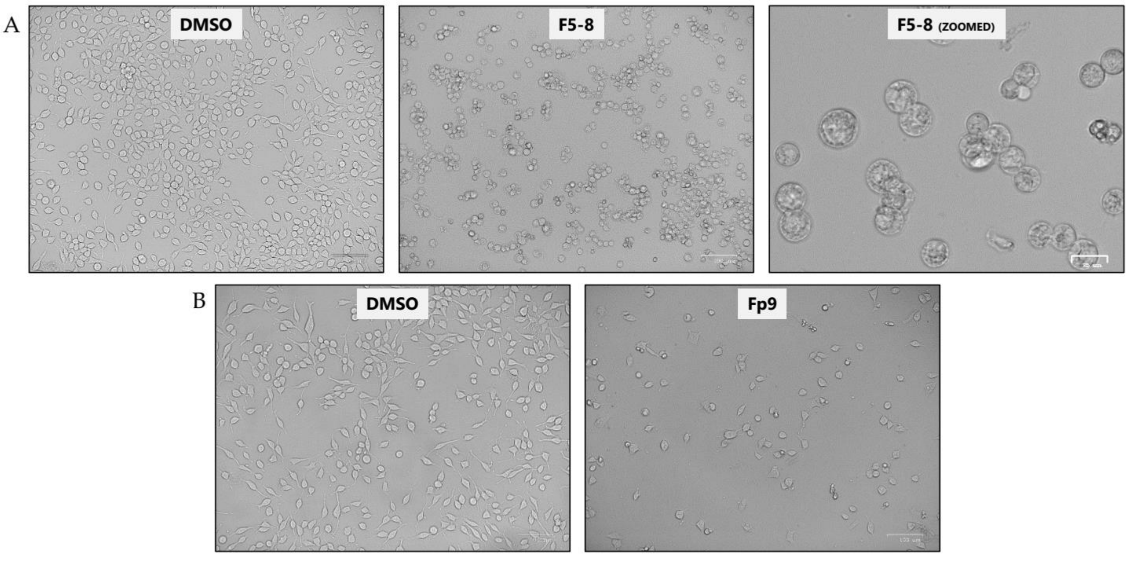

Structural characterization of Fp9 derived from T3 F5-8 (*C. chayamansa*)

Figure S5

GS-MS

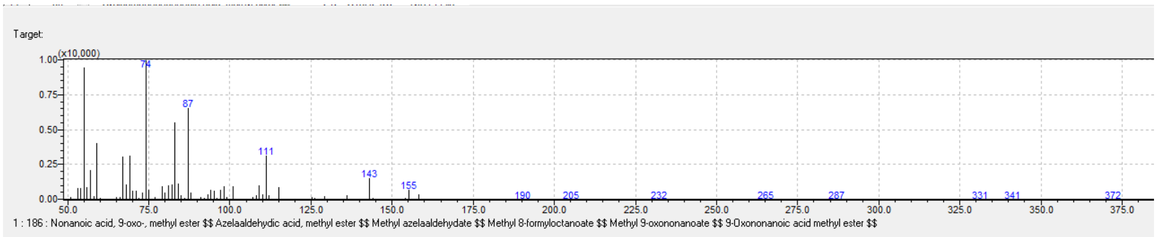

Figure S6.

A. H1 NMR spectroscopy of subfraction Fp9 from T3 F5-8.

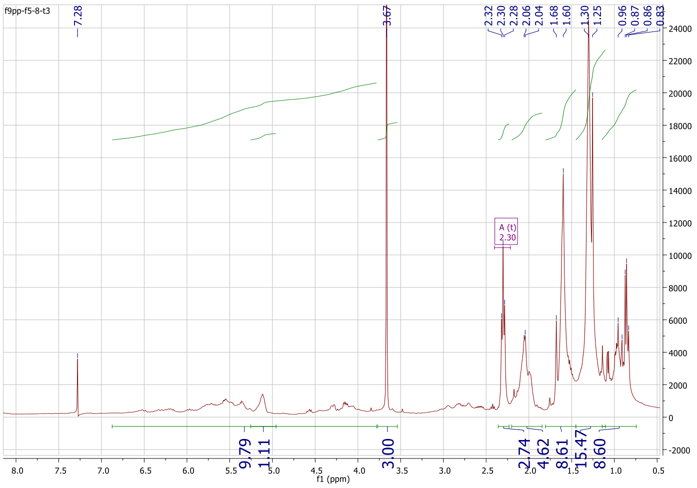

B. Two-dimensional NMR spectroscopy COZY of subfraction Fp9 from T3 F5-8.

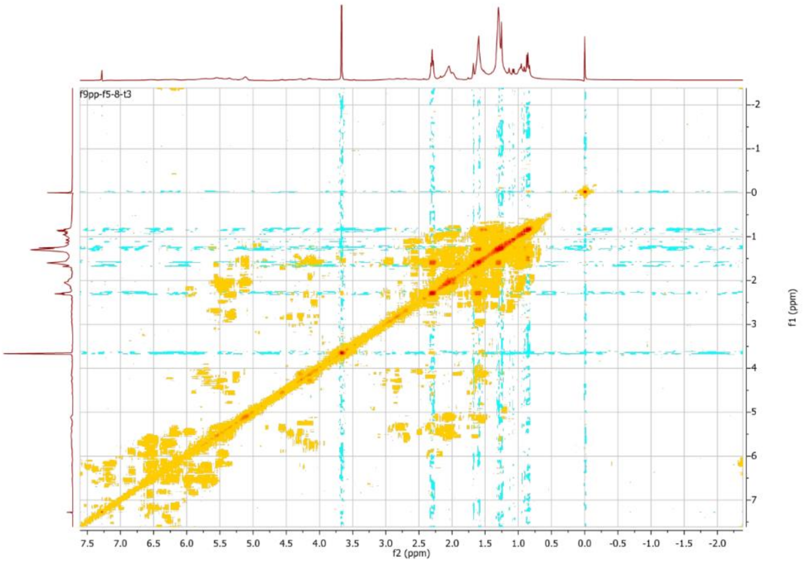

C. Two-dimensional NMR spectroscopy HMBC of subfraction Fp9 from T3 F5-8.

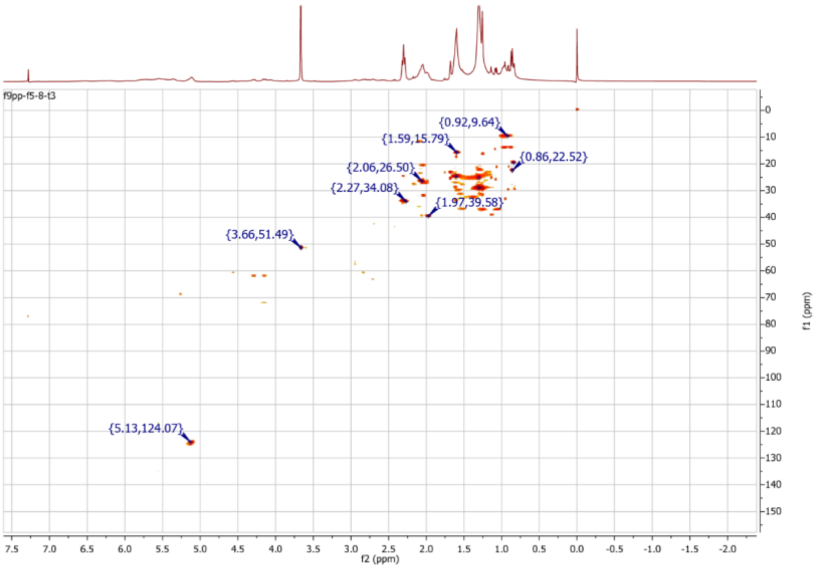

D. Two-dimensional NMR spectroscopy HMBC of subfraction Fp9 from T3 F5-8.

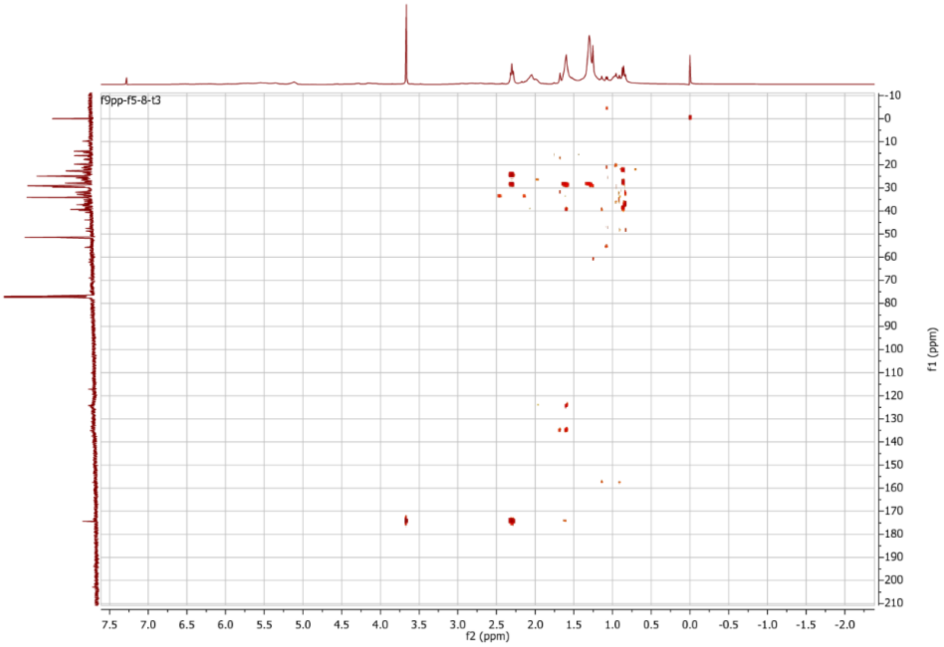

Table S1.
Results of interpretation and peak assignment of the set of NMR techniques.

H[1] δ_H (ppm), integration	Peak multiplicity	*J*H, H (Hz)	Nro	$C^{13}\delta_c$ (ppm)
3.67, 3H	s	-	11	51
-	-	-	1	175
2.30, 2H	t	*J*= 2.32, *J*=2.30, *J*=2.28	2	34.1
2.30, 1H	s		8	28
2.02, 4H	m	-	7 y 5	29 y 39
1.3-1.6	m	-	3,4 y 6	25,29 y 27
1.65, 3H	d	*J*= 1.30, *J*=1.25	9	25
1.28, 3H	d	*J*= 1.30, *J*=1.25	10	25

Figure S7.

Optical microscopy images of cells treated with active fractions of *C. chinense*: A. Representative images of optical microscopy of the LNCaP cells after treated with DMSO (20x), F0 (20x) and F11 (20x) extracts and previously of MTT assay.

A

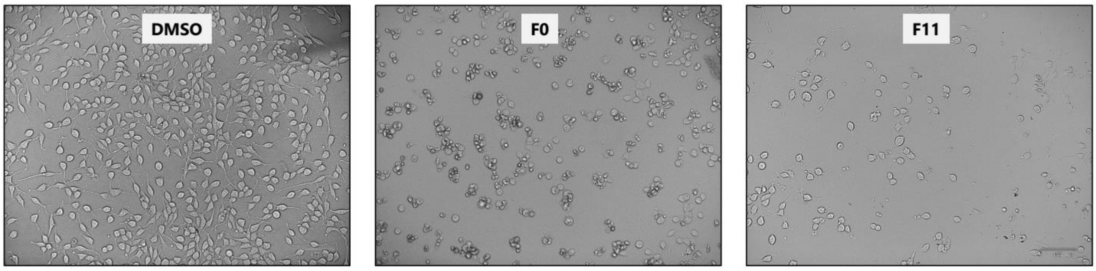

Figure S8.

A. Picture of the preparative chromatography of T31 F0 active fraction of *C. chinense* first column chromatography.

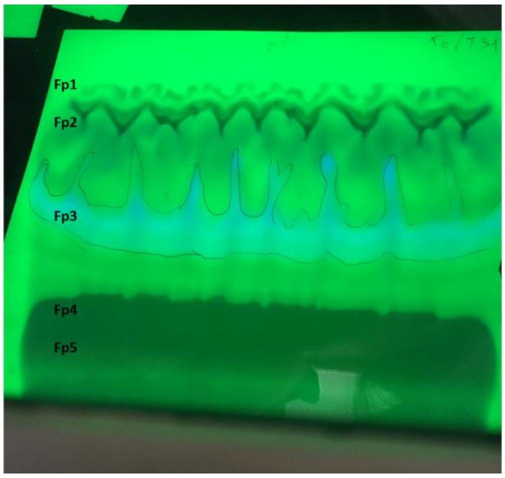

MTT assay of the fraction obtained from the preparative chromatography of F11 (**B**) y F0 (**C**) respectively of *C. chinense*. Statistical significance is *p < 0.05; **p < 0.01.

B

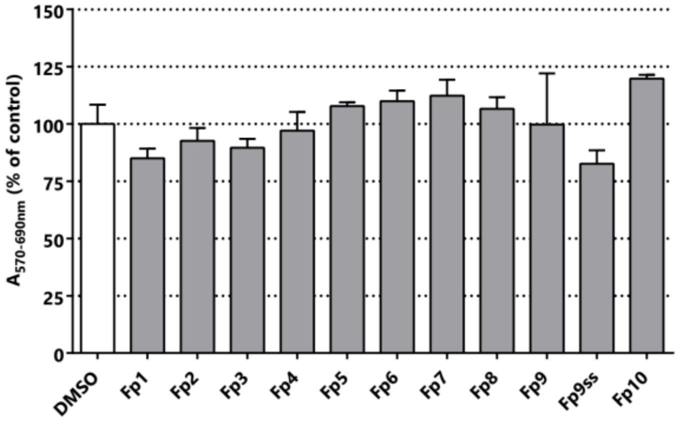

C

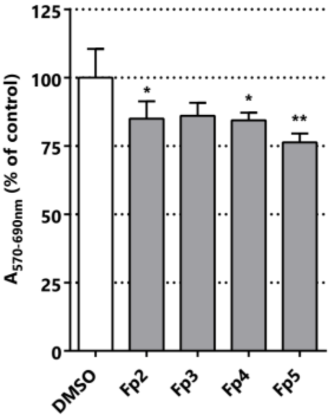

Structural characterization of Fp5 derived from T31 F0 (*C. chinense*)

Figure S9.

A. H NMR spectroscopy of subfraction Fp5 from T31 F0.

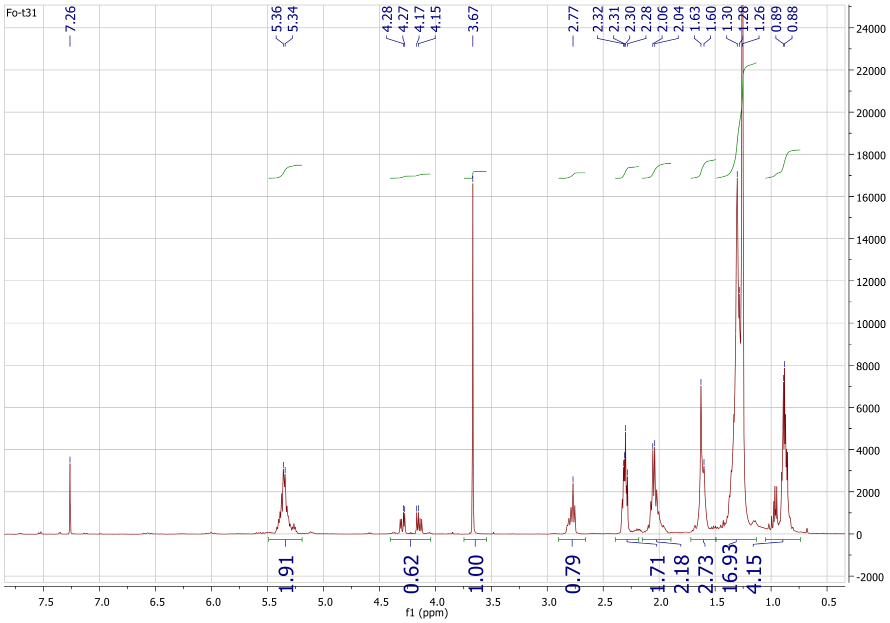

B. Two-dimensional NMR spectroscopy COZY of subfraction Fp5 from T31 F0.

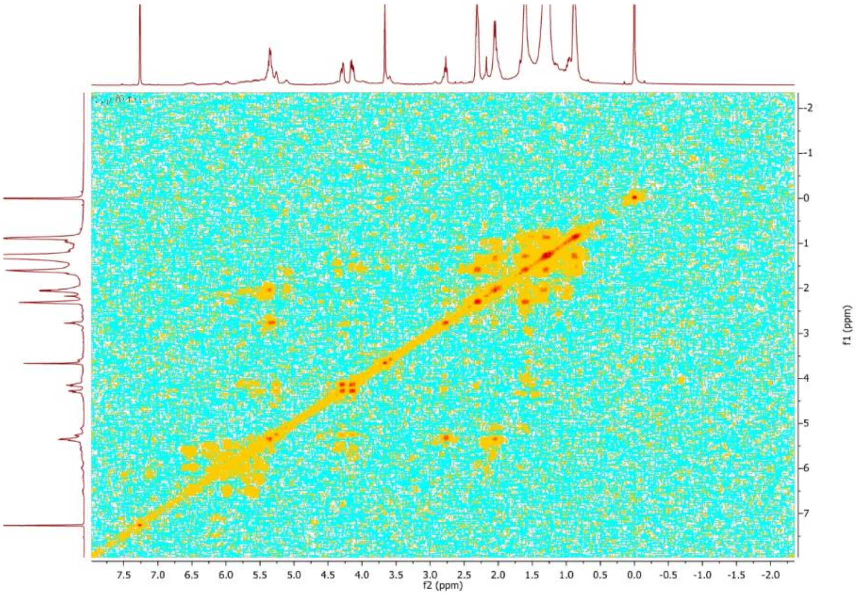

C. Two-dimensional NMR spectroscopy HMBC of subfraction Fp5 from T31 F0.

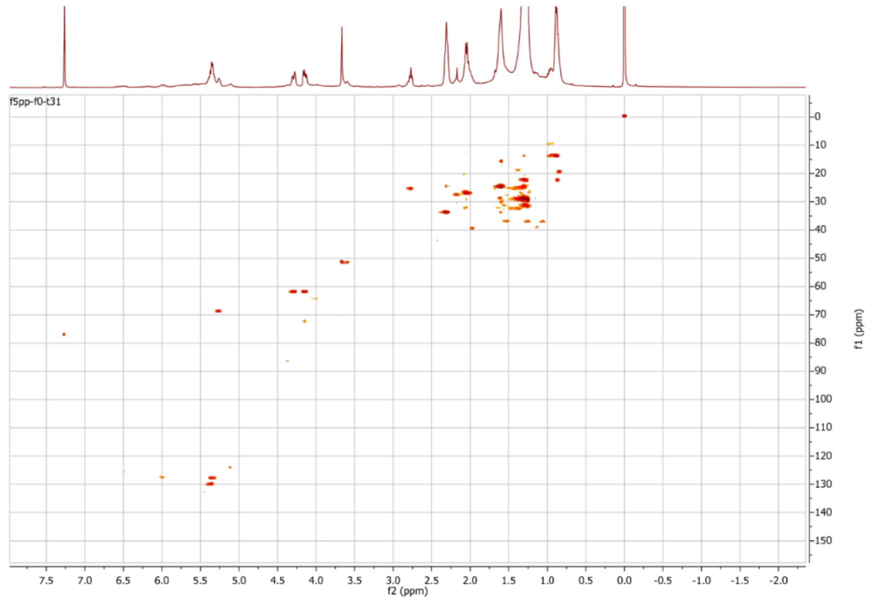

D. Two-dimensional NMR spectroscopy HMBC of subfraction Fp5 from T31 F0.

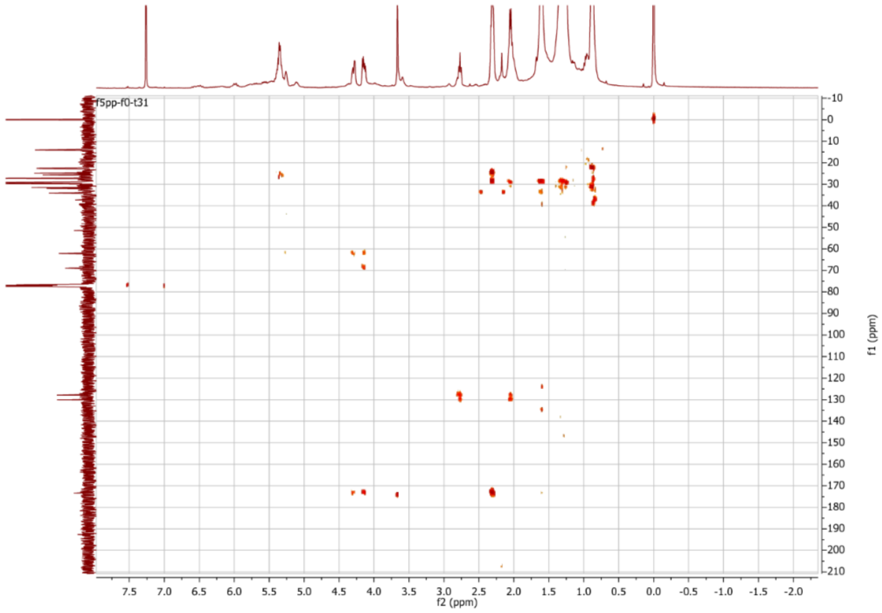

Table S2

Results of interpretation and peak assignment of the set of NMR techniques from T31 F0 Fp5.

H^1 δ_H (ppm), integration	Peak multiplicity	*J*H, H (Hz)	Nro	$C^{13}\delta_c$ (ppm)
-	-	-	1	175
2.30, 2H	m	-	2	34
5.35, 1H	m	-	6	128
5.35, 1H	m	-	7	130
4.28-4.15, 2H	m	-	11	62
2.27, 1H	m	-	3,4,5	32
2.31-2.10, 6H	m	-		25, 29, 32
2.31, 3H	s	-	9	22
2.05, 3H	s	-	10	22

Figure S10.

Structural characterization of Fp9 derived from T31 F11 (*C. chinense*)

A. H NMR spectroscopy of subfraction Fp9 from T31 F11.

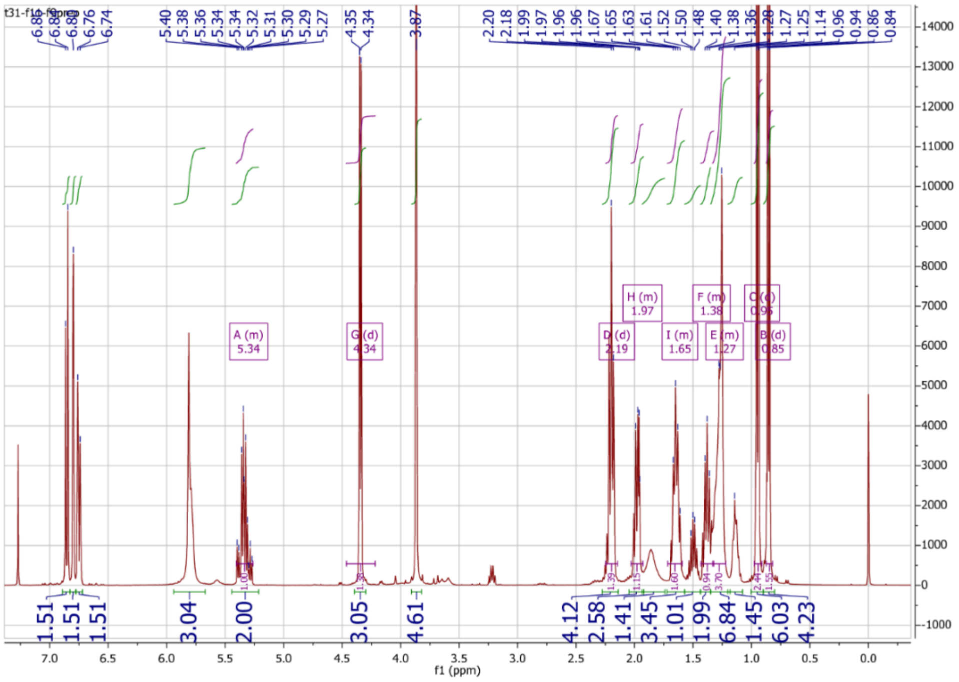

B. Two-dimensional NMR spectroscopy COZY of subfraction Fp9 from T31 F11.

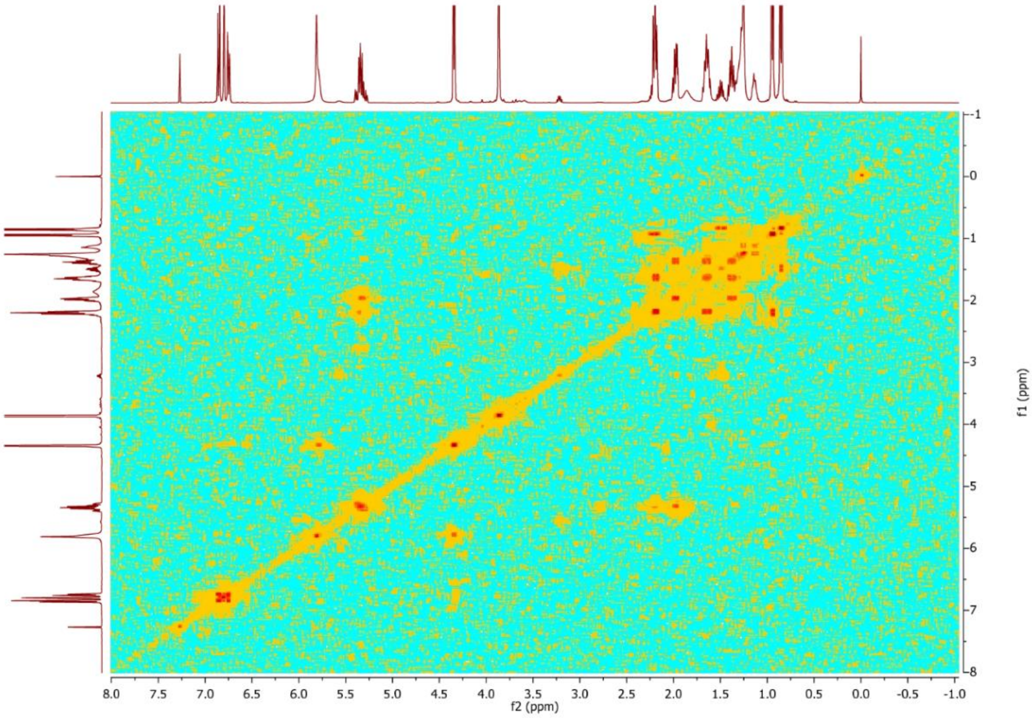

C. Two-dimensional NMR spectroscopy HMBC of subfraction Fp9 from T31 F11.

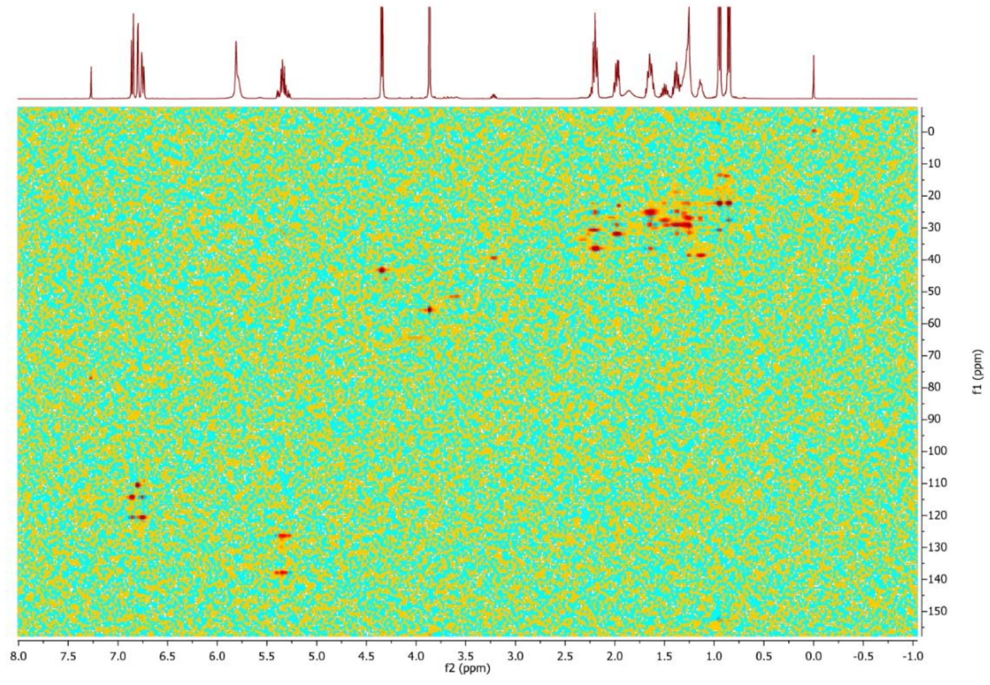

D. Two-dimensional NMR spectroscopy HMBC of subfraction Fp9 from T31 F11.

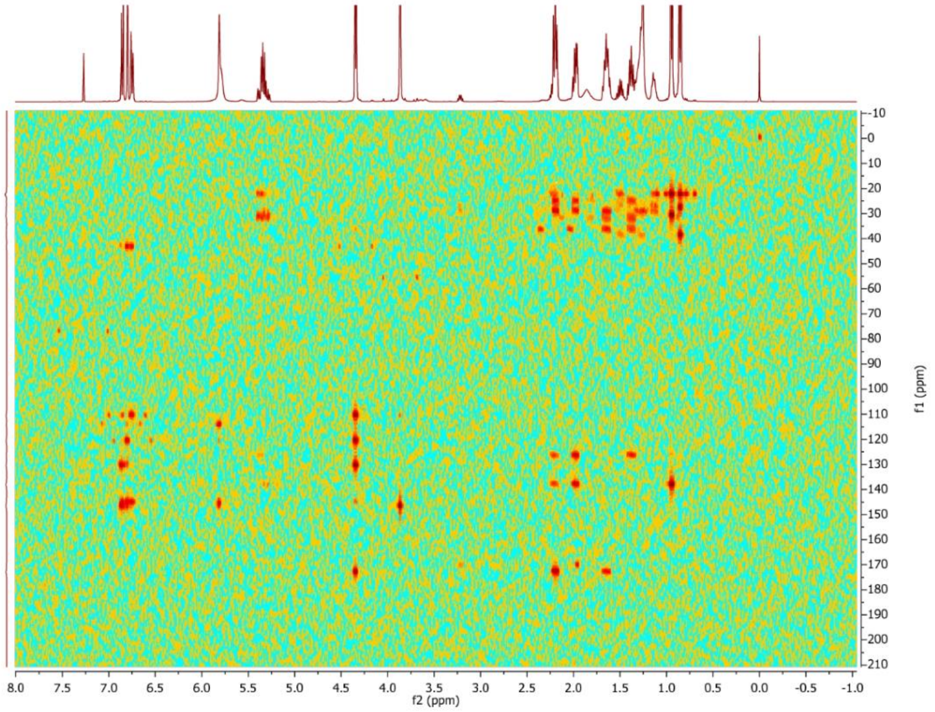

Table S3.

Results of interpretation and peak assignment of the set of NMR techniques from T31 F11 Fp9ss.

H^1 δ_H (ppm), integration	Peak multiplicity	*J*H, H (Hz)	Nro	$C^{13}\delta_c$ (ppm)
-	-	-	*1*	*145.8*
6.80, 1H	*s*	-	*2*	*110.66*
-	-	-	*3*	*120.58*
-	-	-	*4*	*110.3*
6.85, 1H	*dd*	*J=6.86, J=6.84*	*5*	*114.36*
6.75, 1H	*dd*	*J=6.76, J=6.74*	*6*	*120.59*
4.34, 2H	*dd*	*J=4.34, J=4.35*	*7*	*43.45*
-	-	-	*8*	*172.9*
2.19, 2H	*t*	*J=2.22, J=2.20, J=2.18*	*9*	*25.31*

1.65, 2H	*m*	-	*10*	*25.67*
1.38, 2H	*m*	-	*11*	*29.01*
1.97, 2H	*m*	-	*12*	*32.71*
5.33, 1H	*m*	-	*13*	*126.64*
5.33, 1H	*m*	-	*14*	*138.66*
2.19, 1H	*t*	*J=2.22, J=2.20, J=2.18*	*15*	*22.66*
0.95, 3H	*dd*	*J=0.96, J=0.94*	*16*	*22.78*
0.95, 3H	*dd*	*J=0.96, J=0.94*	*17*	*22.78*
3.87, 3H	*s*	-	*18*	*55.46*
5.81, 1H	*s*	-	-	-
5.81, 1H	*s*	-	-	-
